# The use of multiplex PCR for the detection of atypical pathogens in Egyptian children with CAP: a high rate of *Bordetella pertussis* in early infancy

**DOI:** 10.1186/s42506-018-0003-4

**Published:** 2019-01-18

**Authors:** Noussa R. El Basha, Hala H. Shaaban, Hassan A. El Atroush, May M. Sherif, Amani A. El Kholy

**Affiliations:** 1 Department of Pediatrics, Faculty of Medicine, Cairo University, 2 Atteia Abd El Hadi St., El Maadi, Cairo, 11562 Egypt; 2 Department of Clinical Pathology, Faculty of Medicine, Cairo University, 2 Atteia Abd El Hadi St., El Maadi, Cairo, 11562 Egypt

**Keywords:** Community-acquired pneumonia, Children, Atypical pathogen, Pertussis

## Abstract

**Background:**

Atypical pathogen infections played an important role in community-acquired pneumonia (CAP) in children. Pathogen-specific clinical symptoms are often lacking, and it is difficult to detect atypical pathogens by culture methods. The use of multiplex polymerase chain reaction (PCR) methods enables testing for many pathogens simultaneously in a single analysis.

**Aim:**

To determine the role of atypical pathogens in children hospitalized with CAP.

**Patients and methods:**

This cross-sectional study was conducted throughout a 2-year period from August 2015 to September 2017. It included 400 Egyptian children hospitalized with clinical diagnosis of CAP at a tertiary hospital in Cairo, Egypt. Sputum samples were collected from lower respiratory tract of all enrolled patients by mucus trap catheter for identification of *Bordetella pertussis*, *Mycoplasma pneumoniae*, *Chlamydia pneumoniae*, and *Legionella pneumophilia* by using multiplex real-time PCR.

**Results:**

Among the 400 CAP patients enrolled in this study, atypical pathogens were detected in 12/400 (3%) patients. *Bordetella pertussis* was detected in 2% of cases, and it was responsible for CAP in 8/104 (7.69%) infants in the age stratum ≤ 4 months; compared with pertussis-negative cases, pertussis-positive cases were younger and incompletely vaccinated (*P* values were 0.001 and 0.007, respectively). *Mycoplasma pneumoniae* was detected in 1% of cases, all were among the age stratum > 4 months ≤ 59 months in 4/272 (1.47%) children.

**Conclusion:**

In early infancy, *Bordetella pertussis* causes a significant proportion of hospitalized CAP cases; all were ≤ 4 months old and incompletely vaccinated. This finding could suggest the role of maternal immunization in developing countries.

## Introduction

Pneumonia contributes significantly to global childhood morbidity and mortality. The World Health Organization (WHO) estimates that there are 156 million cases of pneumonia each year in children younger than 5 years, with as many as 20 million cases severe enough to require hospital admission [1].

Atypical pneumonia is used to describe pneumonia caused by atypical pathogens such as *Mycoplasma pneumoniae*, *Chlamydia pneumoniae*, *Legionella pneumophili*, and others. The clinical course among children infected with these pathogens is different from that of other bacterial or viral infection, most of them progress slowly and have no specific symptoms. Many studies reported that *Mycoplasma pneumoniae* is the most common atypical pathogen found among children with community-acquired pneumonia (CAP), followed by *Chlamydia pneumoniae* [2–4].

Pertussis (whooping cough) is a respiratory tract infection caused by the Gram-negative bacterium *Bordetella pertussis*. Pertussis is a highly infectious disease and can cause significant morbidity in infants. Recent evidence shows an appreciable incidence of pertussis among infants presenting with acute pneumonia. In a birth cohort, 2% of clinical pneumonia cases among enrolled infants were caused by pertussis [5]. Another study reported that 3.7% of infants and young children presenting to a tertiary academic hospital had evidence of pertussis infection [6]. Most deaths from pertussis occur in developing countries and among children during the first months of life [7]. The WHO estimated that in 2013, *Bordetella pertussis* caused approximately 60,257 deaths in children < 5 years of age [8].

*Mycoplasma pneumoniae* infection shows a variety of clinical manifestations, ranging from asymptomatic infection to fatal pneumonia and may be associated with neurological and systemic symptoms (e.g., rashes). *Mycoplasma pneumoniae* pneumonia has been reported in 10 to 40% of CAP cases, and children are the most susceptible group to mycoplasma infection [9].

The importance of the atypical pathogens as causative agents of pneumonias is not related to their frequency, but to their difficulty of diagnosis and their non-responsiveness to beta-lactam therapy [10]. Atypical bacteria require specific antibiotic coverage because conventional antibiotics used against typical organisms such as streptococci are not effective. Increasing rates of identification of atypical pathogens as a cause of CAP have been reflected in treatment guidelines favoring antibiotics with atypical coverage for certain patients hospitalized with severe CAP [11].

Investigations of typical pathogens causing CAP in children do not include atypical pathogens because it is difficult to detect them by culture methods [2]. On the other hand, multiplex PCR methods enable testing for several pathogens simultaneously in a single analysis.

So, the aim of this study was to determine the role of *Bordetella pertussis*, *Mycoplasma pneumoniae*, *Chlamydia pneumonia*, and *Legionella pneumophilia* in community-acquired pneumonia in children using the real-time PCR as the principal method for determination and also to study the relation, if any, between the presence of these pathogens and clinical, laboratory, and radiological variables.

## Materials and methods

### Study design and subjects

This cross-sectional study was conducted throughout a 2-year period from 1 September 2015 to 31 August 2017. All children fulfilling criteria of case definition of CAP who were hospitalized during this period were enrolled. The study included 400 eligible children, and they were recruited from Emergency Departments of Cairo University Children’s hospitals (The specialized Children Hospital and El Monira Children Hospital), tertiary hospitals that receive patients from all over Egypt. Inclusion criteria were previously healthy children hospitalized with a clinical diagnosis of CAP and patient’s age range from 2 months to 12 years. Exclusion criteria were patients with underlying chronic lung disease, congenital heart disease, immunodeficiency, and chronic hepatic or renal diseases.

## Methods

Data was collected by full-history taking and meticulous clinical examination of all the studied population. All cases were diagnosed to have pneumonia according to WHO case definition of pneumonia [cough or difficult breathing and respiratory rate more than WHO range for age (respiratory rate ≥ 60 breaths/min for children < 2 months; ≥ 50 breaths/min for children 2–11 months; ≥ 40 breaths/min for children 12–59 months)] [12].

CAP is defined as pneumonia that has been acquired in the community in a patient who has not been hospitalized within 14 days prior to the onset of symptoms or has been hospitalized less than 4 days prior to the onset of symptoms [11]. Criteria for classification as severe pneumonia in children include the following: lethargy, unconsciousness, convulsions, inability to drink, vomiting, and lower chest wall indrawing [13].

### Clinical specimens

Complete blood count (CBC) was determined at the time of study enrollment for each patient. Sputum samples (2 ml) were collected from the lower respiratory tract of all enrolled patients by using mucus trap catheter after sputum induction. The samples were transported to the laboratory in an ice container and stored at − 70 °C.

### Molecular identification by real-time PCR

Molecular detection of *Bordetella pertussis*, *Mycoplasma pneumoniae*, *Chlamydia pneumoniae*, and *Legionella pneumophilia* was done by multiplex real-time PCR using AnyplexTM II RB5 Kit (Cat. No. RB7500Y, Seegene, Korea), operated on Biorad CFX 96 real time machine, according to the manufacturer’s instructions [14–16].

Pre-treatment of specimen was done by adding saline solution with the specimen (1:2), vortexed for 1 min, then incubated for 20 min at room temperature.

Conditions and cycles of RT PCR were as follows:

**Table:** 

Segment	No. of cycles	Temperature	Duration
1	1	50 °C	4 min
2	1	95 °C	15 min
3	30	95 °C	30 s
4		60 °C	1 min
5		72 °C	30 s
6	1	55 °C	30 s
7	1	Melting curve 55 ~ 85 °C (5 s/0.5 °C)	
8	10	95 °C	30 s
9		60 °C	1 min
10		72 °C	30 s
11	1	55 °C	30 s
12	1	Melting curve 55 ~ 85 °C (5 s/0.5 °C)	
13	10	95 °C	30 s
14		60 °C	1 min
15		72 °C	30 s
16	1	55 °C	30 s
17	1	Melting curve 55 ~ 85 °C (5 s/0.5 °C)	

RB5 PC-positive control included in the kit was used in each run. It contains mixture of pathogen clones. Also, internal control was added to each sample from the start to ensure high-quality amplification occurred for every sample. The RB5 internal control (IC) controlled not only the nucleic acid isolation procedure but also the possibility of PCR inhibition. Ten microliters of the RB5 IC was added to each specimen before the nucleic acid extraction. Nucleic acid isolation was done using Ribo_spinvRD kit (Cat. No302-150SG1701) from GeneAll.

### Statistical analysis

Data was subjected to computer-assisted statistical analysis using statistical package for social science program IBM SPSS (Statistical Package for the Social Science; IBM Corp, Armonk, NY, USA) release 22 for Microsoft Windows. Nominal data was expressed as frequency and percentage and was compared using chi-square test. Numerical data was expressed as mean ± standard deviation and was compared using *T* test. Non-parametric data was expressed as median “interquartile range” and was compared using Mann-Whitney *U* test. Associations between numerical variables were studied using Pearson’s correlation. *P* values less than 0.05 were considered significant.

### Ethical considerations

This study was approved by Cairo University Clinical Research Ethics Committee, and informed verbal consents were obtained from parents of the included children. The study design conformed to the Revised Helsinki Declaration of Bioethics.

## Results

Among the 400 CAP cases enrolled in this study, atypical pathogens were detected by multiplex PCR in 12/400 (3%) patients. Their mean age was 13.75 ± 15.46 months. Sixty percent of patients were males and 40% were females. A large proportion of the patients had severe pneumonia 104/400 (26%), of them 44 (11%) needed pediatric intensive care unit (PICU) admission. The detailed demographic and clinical characteristics of patients are summarized in Table 1.

**Table 1:** Demographic and clinical characteristics of children with community-acquired pneumonia, Cairo University Specialized Pediatric Hospital

Characteristics	*N* (%)
Age in months (range)	2–84
Mean ± SD	13.75 ± 15.46
Age groups	
≤ 4 months	104 (26%)
> 4 months ≤ 59 months	272 (68%)
> 59 months	24 (6%)
Sex	
Male	240 (60%)
Female	160 (40%)
Incomplete vaccination	200 (50%)
Fever	384 (96%)
Prolonged cough > 14 days	20 (5%)
Chest indrawing	324 (81%)
Rhonchi	76 (19%)
Grunting	64 (16%)
Cyanosis	52 (13%)
Refusal of feeding	104 (26%)
Vomiting	108 (27%)
Lethargy	44 (11%)
Convulsion	24 (6%)
Severe pneumonia	104 (26%)
ICU admission	44 (11%)
Radiological findings	
Unilateral affection	176 (44%)
Bilateral affection	224 (56%)
Number of involved lobes	
One lobe	88 (22%)
Two lobes or more	312 (78%)
Alveolar consolidation	388 (97%)
Consolidation collapse	40 (10%)
Interstitial infiltration	140 (35%)
Pleural effusion	32 (8%)
Total leukocytic count	
Mean ± SD	10.77 ± 5.37
Leukocytosis > 15,000	88 (22%)
Lymphocytosis	80 (20%)
Neutropenia	8 (2%)

Data are described in number of cases (%) and mean ± SD

*Bordetella pertussis* was responsible for CAP in 8/400 (2%) patients, and *Mycoplasma pneumoniae* was determined as the cause of CAP in 4/400 (1%) patients in this study. None were positive for either *C. pneumoniae* or *Legionella pneumophilia* by PCR, as are shown in Table 2.

**Table 2:** The frequency of pathogen detection using the multiplex PCR among the CAP patients

Organism	Number (%)
*Bordetella pertussis*	8 (2%)
*Mycoplasma pneumoniae*	4 (1%)
*Chlamydia pneumoniae*	0 (0%)
*Legionella pneumophilia*	0 (0%)

Data are described in number of cases (%)

*CAP* community-acquired pneumonia

In this work, we did not encounter co-infection by more than one pathogen.

On studying the frequency of pathogen detection among different age stratums, *Bordetella pertussis* was detected in 8/104 (7.69%) infants ≤ 4 months, and pertussis-positive cases were significantly less than 4 months (*P* value ≤ 0.001). All cases of CAP caused by *Mycoplasma pneumonia* were among the age stratum > 4 months ≤ 59 months, as are shown in Table 3.

**Table 3:** Correlating the frequency of pathogen detection to different age groups

Pathogen	Age groups (months)			
	≤ 4 months	> 4 months ≤ 59 months	> 59 months	*P* value
	*n* = 104	*n* = 272	*n* = 24	
*Bordetella pertussis*	8 (7.79%)	0 (0%)	0 (0%)	≤ 0.001
*Mycoplasma pneumoniae*	0 (0%)	4 (1.47%)	0 (0%)	0.3

*P* values > 0.05 is statistically insignificant

Severe pneumonia was recorded in 50% of pertussis-positive patients with no need for PICU admission. Mycoplasma-positive cases did not record severe pneumonia or PICU admission.

On comparing clinical and radiological characteristics of pertussis-positive versus pertussis-negative cases in this study, the percentages of associated upper respiratory symptoms, vomiting, prolonged cough > 14 days, and interstitial infiltration were significantly related to pertussis-positive cases (*P* value was 0.001 for each). Mycoplasma-positive cases were more likely to have unilateral lung involvement with interstitial infiltration compared to mycoplasma-negative cases (*P* value was 0.03 and 0.01, respectively), as are demonstrated in Table 4.

**Table 4:** Comparing *P* value of associated clinical, laboratory, and radiographic findings among positive versus negative pertussis and mycoplasma cases

Variables	Pertussis-positive versus pertussis-negative cases	Mycoplasma-positive versus mycoplasma-negative cases
Prolonged cough > 14 days	≤ 0.001*	> 0.999
Upper respiratory symptoms	0.001*	0.13
Chest indrawing	0.04*	> 0.999
Rhonchi	0.04*	> 0.999
Grunting	0.36	> 0.999
Cyanosis	0.6	> 0.999
Refusal of feeding	0.11	0.57
Vomiting	0.001*	0.57
Lethargy	0.61	> 0.999
Convulsion	1.0	> 0.999
Severe pneumonia	0.1	0.5
ICU admission	0.6	> 0.999
Incomplete vaccination	0.007*	0.12
Radiological findings		
Unilateral affection	0.7	0.03*
Bilateral affection		
Number of involved lobes		
1 lobe	0.08	0.29
2 lobes or more		
Alveolar consolidation	1.0	> 0.999
Interstitial infiltration	0.001*	0.01*
Total leukocytic count		
Leukocytosis > 15,000	0.07	0.58
Lymphocytosis	0.05	0.5

*P values < 0.05 is statistically significant

## Discussion

This study identified atypical pathogens as a potential etiologic agents in community-acquired pneumonia in 12/400 (3%) patients by the use of multiplex PCR. *Bordetella pertussis* was detected in 2% of cases, and it was responsible for CAP in 8/104 (7.69%) infants in the age stratum ≤ 4 months; *Mycoplasma pneumoniae* was detected in 1% of enrolled cases, all were among the age stratum > 4 months ≤ 59 months in 4/272 (1.47%) children.

Compared with pertussis-negative cases, pertussis-positive cases were younger and incompletely vaccinated against *Bordetella pertussis*, and this is in agreement with Sadiasa et al. [17] who found that *Bordetella pertussis* was the most common atypical pneumonia pathogen among hospitalized children. Higher frequencies of pertussis were recorded by Lassmann et al. [18] in Gabon, where they demonstrated that *Bordetella pertussis* caused 6% of cases of CAP in their study; all children who were tested positive for *B. pertussis* were not or only partially immunized. The tendency to infect young unvaccinated or incompletely vaccinated infants suggests the role for maternal pertussis immunization that can significantly increase antibody level for further reduction of disease burden during early infancy.

Pertussis-positive cases in this study were more likely to have been associated with upper respiratory symptoms, vomiting, and prolonged cough > 14 days. Similar results were recorded by Barger-Kamate et al. [19], and this is in concordance with the WHO recommended clinical case definition [20].

In the current study, *Mycoplasma pneumoniae* infection was detected in 1% of cases, which is relatively low, but in keeping with previous studies that reported the detection of *Mycoplasma pneumoniae* by PCR in 1.3% and 4.5% of children aged ≤ 5 years [21, 22]. Higher frequencies of detection of *Mycoplasma pneumoniae* were reported by Defilippi et al. and Hadi et al. who reported 12% and 10% detection rate, respectively [23, 24].

The propensity of *Mycoplasma pneumoniae* detection in the age stratum > 4 months ≤ 59 months in this study could be explained by the mean age of the study population that was 13.75 ± 15.46 months. This finding is supported by Lassmann et al. [18] in Gabon who found that all children who were tested positive for atypical pathogens were less than 5 years old.

Similar to what previously reported by Agarwal et al. [25], this study did not detect *Chlamydia pneumoniae* among cases with CAP. In contrast, Samransamruajkit et al. [26] in Thailand found that *Chlamydia pneumoniae* was responsible for 12.5% of cases of CAP in their study. The available literature supports a low prevalence of *Chlamydia pneumoniae* infection in children younger than 5 years, but it is an important cause of pneumonia among older children [27]. The rate of isolation of *Chlamydia pneumoniae* from acute lower respiratory tract infections in children ranges from 0 to 18% [3, 25, 28–30].

Despite that *Legionella pneumophilia* infection in hospitalized children with CAP has been reported in some studies [2, 31, 32], we did not detect legionella infection among enrolled cases in this study, and this could be explained by our exclusion criteria where we excluded all cases with underling chronic diseases or malignancies. It has been reported that the majority of children with Legionnaires’ disease have an underlying condition such as malignancy [33], renal or liver failure, diabetes, and other conditions that compromise the immune system [34].

There was no significant difference in clinical presentations or TLC between *Mycoplasma pneumoniae*-positive and *Mycoplasma pneumoniae*-negative cases, this is in agreement with Hadi et al. [24] in Iran and Lassmann et al. [18] in Gabon. Also, this was supported by Somer et al. who stated that a clinical diagnosis of infection by atypical bacteria remains elusive [28]. Chest X-ray of mycoplasma-positive cases showed a significant unilateral interstitial infiltration; this is in agreement with other studies [24, 27].

The present study recorded severe pneumonia in 26% of enrolled cases and PICU admission in 11% of cases. Severe pneumonia was encountered in 50% of pertussis-positive patients without the need for PICU admission. At the same time, mycoplasma-positive cases were not associated with severe pneumonia or PICU admission. These findings could be explained by the significantly younger age of pertussis-positive cases. This is consistent with Le Huong et al. [2] in Vietnam, where they found that the highest proportion of severe CAP infections occurred in children younger than 2 years, so the younger the child, the more likely that the atypical pathogen CAP will be severe.

### Limitations of the study

Limiting this study was that its PCR panel did not include other atypical pathogens including respiratory viruses, which are common causes of lower respiratory tract infections. Furthermore, the sample was based on a pre-determined duration rather than calculating a minimum number of cases.

## Conclusion and recommendations

In early infancy, *Bordetella pertussis* caused a significant proportion of hospitalized CAP cases; all were ≤ 4 months old and incompletely vaccinated. This finding could emphasize the role of maternal immunization in developing countries and the importance of complying with the schedule of the DTP vaccine.
